# 6-De­oxy-α-l-talopyran­ose

**DOI:** 10.1107/S1600536808019582

**Published:** 2008-07-05

**Authors:** K. Victoria Booth, Sarah F. Jenkinson, George W. J. Fleet, Pushpakiran Gullapalli, Akihide Yoshihara, Ken Izumori, David J. Watkin

**Affiliations:** a Department of Organic Chemistry, Chemical Research Laboratory, University of Oxford, Mansfield Road, Oxford, OX1 3TA, England; b Rare Sugar Research Centre, Kagawa University, 2393 Miki-cho, Kita-gun, Kagawa 761-0795, Japan; c Department of Chemical Crystallography, Chemical Research Laboratory, University of Oxford, Mansfield Road, Oxford, OX1 3TA, England

## Abstract

X-ray crystallography showed that the title compound, C_6_H_12_O_5_, crystallizes in the α-pyran­ose form with the six-membered ring in a chair conformation. The crystal structure exists as a three-dimensional hydrogen-bonded network of mol­ecules with each mol­ecule acting as a donor and aceptor for four hydrogen bonds. The absolute configuration was determined by the use of l-fucose as starting material.

## Related literature

For related literature, see: Beadle *et al.* (1992 ▶); Izumori (2002 ▶, 2006 ▶); Granstrom *et al.* (2004 ▶); Yoshihara *et al.* (2008 ▶).
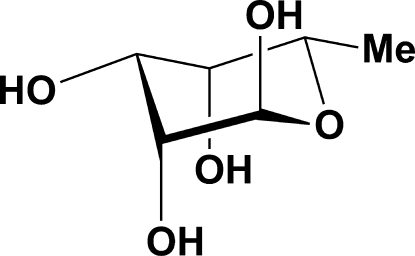


## Experimental

### 

#### Crystal data

C_6_H_12_O_5_
*M* *_r_* = 164.16
Orthorhombic, 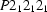

*a* = 6.4939 (3) Å
*b* = 7.4874 (4) Å
*c* = 14.8382 (8) Å
*V* = 721.47 (6) Å^3^
*Z* = 4
Mo *K*α radiation
μ = 0.13 mm^−1^
*T* = 150 K
0.25 × 0.25 × 0.02 mm

#### Data collection

Nonius KappaCCD diffractometer
Absorption correction: multi-scan *DENZO*/*SCALEPACK* (Otwinowski & Minor, 1997 ▶) *T*_min_ = 0.97, *T*_max_ = 1.00 (expected range = 0.967–0.997)
4390 measured reflections
968 independent reflections
863 reflections with *I* > 2.0σ(*I*)
*R*_int_ = 0.037

#### Refinement

*R*[*F* ^2^ > 2σ(*F* ^2^)] = 0.029
*wR*(*F* ^2^) = 0.072
*S* = 1.03
968 reflections
100 parameters
H-atom parameters constrained
Δρ_max_ = 0.24 e Å^−3^
Δρ_min_ = −0.21 e Å^−3^

### 

Data collection: *COLLECT* (Nonius, 1997–2001 ▶).; cell refinement: *DENZO*/*SCALEPACK* (Otwinowski & Minor, 1997 ▶); data reduction: *DENZO*/*SCALEPACK*; program(s) used to solve structure: *SIR92* (Altomare *et al.*, 1994 ▶); program(s) used to refine structure: *CRYSTALS* (Betteridge *et al.*, 2003 ▶); molecular graphics: *CAMERON* (Watkin *et al.*, 1996 ▶); software used to prepare material for publication: *CRYSTALS*.

## Supplementary Material

Crystal structure: contains datablocks global, I. DOI: 10.1107/S1600536808019582/lh2652sup1.cif

Structure factors: contains datablocks I. DOI: 10.1107/S1600536808019582/lh2652Isup2.hkl

Additional supplementary materials:  crystallographic information; 3D view; checkCIF report

## Figures and Tables

**Table 1:** Hydrogen-bond geometry (Å, °)

*D*—H⋯*A*	*D*—H	H⋯*A*	*D*⋯*A*	*D*—H⋯*A*
O9—H7⋯O1^i^	0.81	2.04	2.818 (2)	162
O1—H8⋯O10	0.82	1.98	2.740 (2)	156
O10—H10⋯O9^i^	0.84	1.85	2.686 (2)	177
O11—H1⋯O4^ii^	0.87	1.94	2.812 (2)	177

Symmetry codes: (i) 
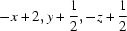
; (ii) 
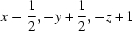
.

**Fig. 1.**
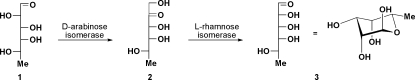
Synthetic scheme.

**Fig. 2.**
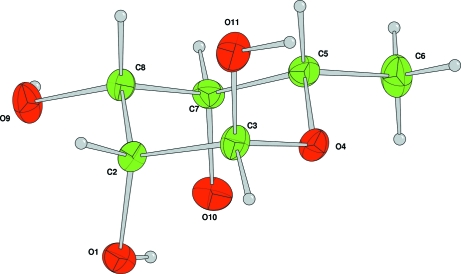
The title compound with displacement ellipsoids drawn at the 50% probability level. H atoms are shown as spheres of arbitary radius.

**Fig. 3.**
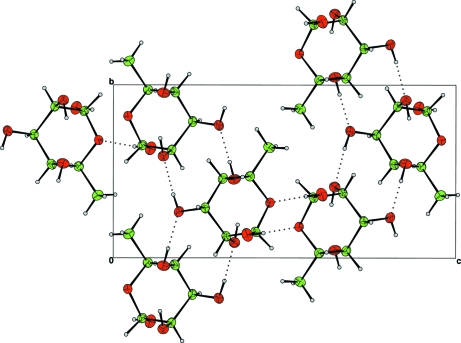
Packing diagram of the title compound projected along the a-axis.

## supplementary crystallographic information

### Comment

The range of rare sugars that are now readily available has increased in recent years due to both chemical (Beadle *et al.*, 1992) and biotechnological (Izumori, 2006; Izumori, 2002; Granstrom *et al.*, 2004) advances. The methodology developed by Izumori *et al.* for the interconversion of tetroses, pentoses and hexoses by enzymatic oxidation, inversion at C3 with a single epimerase, and reduction to the aldose has been seen to be generally applicable for the 1-deoxy ketohexoses (Yoshihara *et al.*, 2008) in large amounts in water.

The Izumoring method is demonstrated here with the synthesis of 6-deoxy-*L*-talose 3 from *L*-fucose 1(Fig. 1) by a series of isomerizations. Firstly, using D-arabinose isomerase, *L*-fucose was isomerized to 6-deoxy-*L*-tagatose 2 and then using *L*-rhamnose isomerase this was further isomerized to give 6-deoxy-*L*-talose 3.

6-Deoxy-*L*-talose crystallizes solely in the α-pyranose form (Fig. 2). The absolute configuration was determined from the starting material. The crystal exists as an hydrogen bonded network with each molecule acting as a donor and acceptor for 4 hydrogen bonds. Non-conventional hydrogen bonds have been ignored.

### Experimental

The title compound was recrystallized from methanol: m.p. 120–123°C; [α]_D_^20^ -18.6 (*c*, 0.94 in H_2_O).

### Refinement

In the absence of significant anomalous scattering, Friedel pairs were merged and the absolute configuration was assigned from the starting material.

The H atoms were all located in a difference map, but those attached to carbon atoms were repositioned geometrically. The H atoms were initially refined with soft restraints on the bond lengths and angles to regularize their geometry (C—H in the range 0.93–0.98, O—H = 0.82 Å) and *U*_iso_(H) (in the range 1.2–1.5 times *U*_eq_ of the parent atom), after which the positions were refined with riding constraints.

### Crystal data

**Table:**

C_6_H_12_O_5_	*F*_000_ = 352
*M**_r_* = 164.16	*D*_x_ = 1.511 Mg m^−^^3^
Orthorhombic, *P*2_1_2_1_2_1_	Mo *K*α radiation λ = 0.71073 Å
Hall symbol: P 2ac 2ab	Cell parameters from 890 reflections
*a* = 6.4939 (3) Å	θ = 5–27º
*b* = 7.4874 (4) Å	µ = 0.13 mm^−^^1^
*c* = 14.8382 (8) Å	*T* = 150 K
*V* = 721.47 (6) Å^3^	Plate, colourless
*Z* = 4	0.25 × 0.25 × 0.02 mm

### Data collection

**Table:**

Nonius KappaCCD diffractometer	863 reflections with *I* > 2.0σ(*I*)
Monochromator: graphite	*R*_int_ = 0.037
*T* = 150 K	θ_max_ = 27.5º
ω scans	θ_min_ = 5.2º
Absorption correction: multi-scanDENZO/SCALEPACK (Otwinowski & Minor, 1997)	*h* = −8→8
*T*_min_ = 0.97, *T*_max_ = 1.00	*k* = −9→9
4390 measured reflections	*l* = −19→19
968 independent reflections	

### Refinement

**Table:**

Refinement on *F*^2^	Hydrogen site location: inferred from neighbouring sites
Least-squares matrix: full	H-atom parameters constrained
*R*[*F*^2^ > 2σ(*F*^2^)] = 0.029	Method = Modified Sheldrick *w* = 1/[σ^2^(*F*^2^) + ( 0.04*P*)^2^ + 0.04*P*] ,where *P* = (max(*F*_o_^2^,0) + 2*F*_c_^2^)/3
*wR*(*F*^2^) = 0.072	(Δ/σ)_max_ = 0.0002
*S* = 1.03	Δρ_max_ = 0.24 e Å^−^^3^
968 reflections	Δρ_min_ = −0.21 e Å^−^^3^
100 parameters	Extinction correction: None
Primary atom site location: structure-invariant direct methods	

### Fractional atomic coordinates and isotropic or equivalent isotropic displacement parameters (Å^2^)

**Table:**

	*x*	*y*	*z*	*U*_iso_*/*U*_eq_	
O1	0.95002 (16)	0.08900 (18)	0.35376 (7)	0.0210	
C2	0.7416 (2)	0.1250 (2)	0.32982 (10)	0.0175	
C3	0.6113 (3)	0.1509 (3)	0.41551 (10)	0.0188	
O4	0.65011 (17)	0.31964 (16)	0.45740 (7)	0.0183	
C5	0.6150 (2)	0.4717 (2)	0.39897 (10)	0.0194	
C6	0.6412 (3)	0.6382 (3)	0.45454 (12)	0.0271	
C7	0.7657 (2)	0.4593 (2)	0.32003 (10)	0.0187	
C8	0.7208 (2)	0.2889 (3)	0.26846 (10)	0.0186	
O9	0.85143 (18)	0.26675 (18)	0.19178 (7)	0.0243	
O10	0.97297 (17)	0.45445 (18)	0.35319 (8)	0.0232	
O11	0.40458 (18)	0.1333 (2)	0.39115 (8)	0.0254	
H21	0.6937	0.0193	0.2985	0.0197*	
H31	0.6474	0.0567	0.4621	0.0197*	
H51	0.4756	0.4675	0.3773	0.0236*	
H61	0.6179	0.7441	0.4188	0.0406*	
H62	0.5480	0.6346	0.5057	0.0398*	
H63	0.7791	0.6452	0.4743	0.0401*	
H71	0.7508	0.5628	0.2786	0.0214*	
H81	0.5761	0.2938	0.2475	0.0221*	
H7	0.8826	0.3664	0.1753	0.0374*	
H8	0.9866	0.1914	0.3632	0.0333*	
H10	1.0243	0.5538	0.3399	0.0350*	
H1	0.3258	0.1436	0.4383	0.0408*	

### Atomic displacement parameters (Å^2^)

**Table:**

	*U*^11^	*U*^22^	*U*^33^	*U*^12^	*U*^13^	*U*^23^
O1	0.0198 (5)	0.0204 (7)	0.0229 (6)	0.0030 (5)	−0.0010 (5)	−0.0005 (5)
C2	0.0184 (7)	0.0188 (9)	0.0153 (7)	−0.0001 (8)	−0.0013 (6)	−0.0015 (6)
C3	0.0209 (8)	0.0186 (9)	0.0169 (7)	−0.0037 (7)	0.0005 (6)	−0.0021 (7)
O4	0.0223 (5)	0.0178 (7)	0.0150 (5)	−0.0008 (5)	−0.0006 (5)	0.0007 (5)
C5	0.0220 (8)	0.0184 (9)	0.0179 (7)	0.0019 (8)	−0.0015 (7)	0.0002 (7)
C6	0.0378 (10)	0.0202 (10)	0.0232 (8)	0.0022 (9)	0.0025 (9)	−0.0040 (7)
C7	0.0183 (7)	0.0202 (9)	0.0176 (7)	0.0031 (7)	0.0006 (7)	0.0029 (7)
C8	0.0197 (7)	0.0212 (10)	0.0149 (7)	0.0011 (7)	0.0013 (6)	−0.0005 (7)
O9	0.0335 (6)	0.0213 (7)	0.0180 (5)	0.0043 (6)	0.0087 (5)	0.0025 (5)
O10	0.0195 (5)	0.0195 (7)	0.0306 (6)	−0.0026 (5)	−0.0015 (5)	0.0023 (6)
O11	0.0199 (5)	0.0331 (8)	0.0233 (5)	−0.0074 (6)	0.0028 (5)	−0.0047 (6)

### Geometric parameters (Å, °)

**Table:**

O1—C2	1.4250 (19)	C6—H61	0.966
O1—H8	0.815	C6—H62	0.971
C2—C3	1.540 (2)	C6—H63	0.944
C2—C8	1.534 (2)	C7—C8	1.516 (2)
C2—H21	0.969	C7—O10	1.4334 (19)
C3—O4	1.430 (2)	C7—H71	0.994
C3—O11	1.3965 (19)	C8—O9	1.4288 (18)
C3—H31	1.015	C8—H81	0.990
O4—C5	1.449 (2)	O9—H7	0.811
C5—C6	1.504 (2)	O10—H10	0.839
C5—C7	1.529 (2)	O11—H1	0.870
C5—H51	0.962		
			
C2—O1—H8	98.1	C5—C6—H62	109.6
O1—C2—C3	109.88 (12)	H61—C6—H62	110.8
O1—C2—C8	112.50 (13)	C5—C6—H63	108.9
C3—C2—C8	109.93 (14)	H61—C6—H63	105.9
O1—C2—H21	105.7	H62—C6—H63	110.5
C3—C2—H21	108.8	C5—C7—C8	108.34 (14)
C8—C2—H21	109.9	C5—C7—O10	109.84 (12)
C2—C3—O4	111.93 (14)	C8—C7—O10	109.43 (14)
C2—C3—O11	107.62 (12)	C5—C7—H71	111.3
O4—C3—O11	111.43 (15)	C8—C7—H71	109.0
C2—C3—H31	110.4	O10—C7—H71	108.9
O4—C3—H31	106.1	C2—C8—C7	110.89 (12)
O11—C3—H31	109.4	C2—C8—O9	109.12 (13)
C3—O4—C5	113.97 (11)	C7—C8—O9	112.67 (13)
O4—C5—C6	107.79 (12)	C2—C8—H81	107.4
O4—C5—C7	108.05 (13)	C7—C8—H81	108.0
C6—C5—C7	113.45 (14)	O9—C8—H81	108.5
O4—C5—H51	108.8	C8—O9—H7	106.4
C6—C5—H51	108.5	C7—O10—H10	105.7
C7—C5—H51	110.1	C3—O11—H1	110.4
C5—C6—H61	111.2		

### Hydrogen-bond geometry (Å, °)

**Table:**

*D*—H···*A*	*D*—H	H···*A*	*D*···*A*	*D*—H···*A*
O9—H7···O1^i^	0.81	2.04	2.818 (2)	162
O1—H8···C7	0.82	2.55	3.061 (2)	122
O1—H8···O10	0.82	1.98	2.740 (2)	156
O10—H10···O9^i^	0.84	1.85	2.686 (2)	177
O11—H1···O4^ii^	0.87	1.94	2.812 (2)	177

Symmetry codes: (i) −*x*+2, *y*+1/2, −*z*+1/2; (ii) *x*−1/2, −*y*+1/2, −*z*+1.
